# Enhanced Superconductivity and Rashba Effect in a Buckled Plumbene‐Au Kagome Superstructure

**DOI:** 10.1002/advs.202300845

**Published:** 2023-05-03

**Authors:** Wan‐Hsin Chen, Chin‐Hsuan Chen, Guan‐Hao Chen, Wei‐Chuan Chen, Fu‐Xiang Rikudo Chen, Pei‐Jung Chen, Chun‐Kai Ku, Chang‐Tsan Lee, Naoya Kawakami, Jia‐Ying Li, Iwao Matsuda, Wen‐Hao Chang, Juhn‐Jong Lin, Chien‐Te Wu, Chung‐Yu Mou, Horng‐Tay Jeng, Shu‐Jung Tang, Chun‐Liang Lin

**Affiliations:** ^1^ Department of Electrophysics National Yang Ming Chiao Tung University Hsinchu 300 Taiwan; ^2^ Center for Quantum Technology and Department of Physics National Tsing Hua University Hsinchu 300 Taiwan; ^3^ Research Center for Applied Sciences Academia Sinica Taipei 115 Taiwan; ^4^ Center for Emergent Functional Matter Science National Yang Ming Chiao Tung University Hsinchu 300 Taiwan; ^5^ Institute for Solid State Physics The University of Tokyo Kashiwa 277‐8568 Japan; ^6^ Physics Division National Center for Theoretical Sciences Taipei 106 Taiwan; ^7^ Institute of Physics Academia Sinica Taipei 115 Taiwan; ^8^ National Synchrotron Radiation Research Center Hsinchu 300 Taiwan

**Keywords:** angle‐resolved photoemission spectroscopy, buckled plumbene, density functional theory, electron–phonon coupling, Rashba effect, scanning tunneling microscopy, superconductivity

## Abstract

Plumbene, with a structure similar to graphene, is expected to possess a strong spin–orbit coupling and thus enhances its superconducting critical temperature (*T*_c_). In this work, a buckled plumbene‐Au Kagome superstructure grown by depositing Au on Pb(111) is investigated. The superconducting gap monitored by temperature‐dependent scanning tunneling microscopy/spectroscopy shows that the buckled plumbene‐Au Kagome superstructure not only has an enhanced *T*_c_ with respect to that of a monolayer Pb but also possesses a higher value than what owned by a bulk Pb substrate. By combining angle‐resolved photoemission spectroscopy with density functional theory, the monolayer Au‐intercalated low‐buckled plumbene sandwiched between the top Au Kagome layer and the bottom Pb(111) substrate is confirmed and the electron–phonon coupling‐enhanced superconductivity is revealed. This work demonstrates that a buckled plumbene‐Au Kagome superstructure can enhance superconducting *T*_c_ and Rashba effect, effectively triggering the novel properties of a plumbene.

## Graphical abstract

The enhanced superconductivity in a buckled plumbene‐Au Kagome structure is observed by scanning tunneling microscopy/spectroscopy. The monolayer Au‐intercalated buckled plumbene is sandwiched between a top Au Kagome layer and the Pb(111) substrate, as confirmed by the measured and calculated electronic structures. Further analysis reveals a significant Rashba effect and electron–phonon coupling‐enhanced critical temperature.

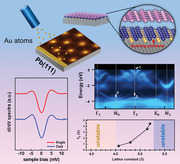

## 1 Introduction

Two dimensional (2D) superconducting layers have attracted considerable attention because the reduction of dimensionality opens to explore unconventional quantum phase transition in strongly correlated systems.^[^ ^1^, ^2^ ^]^ Several works have studied the properties of 2D superconducting systems, including metal superlattice on semiconductor substrates, monolayer sheet in van der Waals stacking, organic molecule films, the interface between oxides, etc.^[^ ^3^, ^4^ ^]^ Especially for current research on van der Waals materials such as NbSe_2_, FeSe,^[^ ^4^ ^]^ and twisted bilayer graphene^[^ ^3^ ^]^ those are going to arise the possibility of using 2D layers to achieve ultrathin superconductors. In addition, 2D superconductors made of heavy elements, such as Pb or In, can provide a fascinating platform for studying topological superconductivity^[^ ^5^ ^]^ and tracing novel quasiparticles such as Majorana fermions.^[^ ^6^, ^7^ ^]^ In a 2D system, when the translational symmetry is broken, the spatial reversal symmetry is also forbidden. Therefore, the spin–orbit coupling (SOC), which spontaneously arises from the lack of inversion symmetry becomes influential. The most typical example is the Rashba effect.^[^ ^8^ ^]^ The Rashba effect lifts spin degeneracy in a time‐reversal invariant system with broken translational symmetry, which leads to many exotic quantum phenomena and novel applications.^[^ ^9^, ^10^, ^11^ ^]^ Moreover, a theoretical prediction shows that the Rashba SOC influences superconducting pairing as well as the density of states (DOS) to enhance the superconducting critical temperature (*T*_c_).^[^ ^12^ ^]^

Pb thin films exhibit superconducting *T*_c_,^[^ ^13^, ^14^, ^15^ ^]^ ≈6 K, and the *T*_c_ changes with film thickness because of the modulated DOS near the Fermi level (*E*_F_) by the quantum size effect. However, the *T*_c_’s rapidly decreasing to less than 2 K as the film thickness is reduced^[^ ^14^ ^]^ to monoatomic layer leads to a limitation for further application. Plumbene, which is a monolayer honeycomb consisting of Pb and in a form different from typical monolayer Pb may have a potential to enhance *T*_c_ when it takes a buckled configuration in between sp^2^ and sp^3^ hybridizations. Similarly to other honeycomb lattices consisting of the elements in group 14, such as silicene,^[^ ^16^, ^17^ ^]^ germanene,^[^ ^18^, ^19^ ^]^ stanene,^[^ ^20^ ^]^ plumbene has been successfully fabricated on Pd(111)^[^ ^21^ ^]^ and Fe/Ir(111)^[^ ^22^ ^]^ surfaces. The buckled configuration with increasing atomic weight that provides relevant SOC can induce many exotic properties.^[^ ^23^, ^24^, ^25^, ^26^, ^27^ ^]^ Plumbene was also predicted to enhance *T*_c_ in a high‐buckled configuration.^[^ ^28^ ^]^ However, a experimental confirmation is still lacking. On the other hand, the low‐buckled plumbene has been extensively studied, yet no superconductivity has been reported to date. Moreover, with the high *Z* value of a Pb atom, namely SOC, plumbene was also expected to have a promising quantum spin Hall effect.^[^ ^29^ ^]^ Combing buckled plumbene and SOC can lead to a candidate of 2D topological superconductor. Meanwhile, transition metal Kagome structure hosting unconventional charge orders can lead to strongly correlated quantum phase such as superconductivity and pair density wave in AV_3_Sb_5_ family (A = K, Rb or Cs).^[^ ^30^, ^31^ ^]^

Doping or intercalating with foreign atoms, and even fabricating twisted bilayer structures, which have been widely applied methods to tune the graphene and 2D materials properties,^[^ ^32^, ^33^ ^]^ were theoretically employed to study the tunability of plumbene. On the other hand, binary surface alloys were widely known to exhibit relevant Rashba effect.^[^ ^34^, ^35^ ^]^ Among them, Pb_2_Au alloy system was discovered experimentally to exhibit large Rashba strength and a model of a moderately buckled Pb_2_Au alloy sandwiched between a top Kagome layer and the bottom Pb(111) was proposed in collaboration with density functional theory (DFT) calculation to elucidate the Rashba effect.^[^ ^35^ ^]^ The single Pb_2_Au layer can likely be regarded as a low‐buckled plumbene with Au atoms inserted into the hollow centers of honeycombs, and thus will be named Au‐plumbene in this work. With this thought and the certified Rashba effect, it is interesting to probe the superconductivity of Au‐plumbene/Au Kagome superstructure. If enhanced *T*_c_ value is detected, an experimentally feasible way to trigger the novel property of plumbene is manifested. By combining the experiment of temperature‐dependent scanning tunneling spectroscopy (STS), angle‐resolved photoemission spectroscopy (ARPES), and DFT calculation, we detected an enhanced *T*_c_ with respect to Pb(111) and unraveled the spin texture of band structures of the Au‐plumbene/Au Kagome superstructure. The electron–phonon coupling (EPC) enhanced superconductivity of this system is further explained by the calculation on the model of a buckled‐height dependent Au‐plumbene layer.

## 2 Results and Discussion

### 2.1 Observation of Superconductivity in Buckled Pb_2_Au–Au Kagome Superstructure

**Figure 1:**
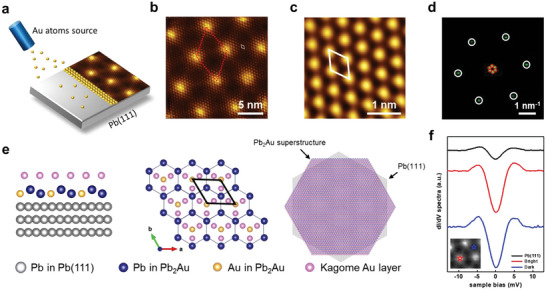
Superconductive properties of buckled Pb_2_Au–Au Kagome superstructure. a) The schematic diagram of preparation setup of buckled Pb_2_Au–Au Kagome superstructure. b) STM topographic images of a distinct moiré pattern on the surface. Image size is 20 × 20 nm^2^ (*V* = 0.2 V and *I* = 1.0 nA). c) Small 1 × 1 atomic configuration on the surface. Image size is 3 × 3 nm^2^ (*V* = 0.2 V and *I* = 1.0 nA). d) Corresponding FFT of b). e) The schematic atomic model of Pb_2_Au superstructure on Pb(111) substrate. Left, the side view of the sandwiched Pb_2_Au superstructure system; middle, the top view of Pb_2_Au superstructure, the topmost Au layer is on top of the buckled Pb_2_Au in the middle and forms the Kagome structure; right, the schematic diagram of moiré pattern. f) STS spectra acquired on Pb(111) and Pb_2_Au superstructure at 4.8 K. The star marks in the inset figure point out the bright and dark regions on the Pb_2_Au superstructure.

The schematic setup for the sample preparation in **Figure** **1**a shows Au atoms were evaporated onto a Pb(111) surface from an effusion cell held at ≈1050 °C in an ultrahigh vacuum. Figure 1b shows a representative scanning tunneling microscopy (STM) topographic image of a distinct moiré pattern on the surface coexisting with hexagonal domains together with the fundamental 1 × 1 atomic configuration. As shown in Figure 1b and the zoomed‐in STM image in Figure 1c, the unit cell of the moiré pattern, represented by the red dot line, appears with a period of 65.6 ± 0.5 Å; the unit cell of the 1 × 1 atomic configuration, represented by the white line, appears with a lattice constant of 5.7 ± 0.1 Å, respectively. Figure 1d shows the 2D Fast Fourier transform (FFT) of Figure 1b. The inner six spots, marked by red circles, correspond to the moiré pattern, and the outer six spots, marked by white circles, represent the basic 1 × 1 atomic configuration, respectively. The moiré pattern exhibits a rotation by 30° with respect to the small 1 × 1 atomic configuration, and additionally, the small 1 × 1 atomic configuration consisting of Kagome Au layer is attached to the Pb_2_Au layer accordingly.^[^ ^35^, ^36^ ^]^ Interestingly, the period of the moiré pattern is different from the values observed in previous studies.^[^ ^37^ ^]^ At this stage, we have to consider the twist angles as suggested by both the Pb_2_Au superstructure and the underlying Pb(111) layer, as discussed later.

The sandwiched atomic model of the buckled Pb_2_Au–Au Kagome superstructure system (simplified as Pb_2_Au superstructure later) is shown in Figure 1e. The bulk Pb(111) layer, represented by the gray balls, was used as a substrate for Pb_2_Au superstructure. The blue and yellow balls represent the lead and gold atoms of the Pb_2_Au in the middle layer, respectively. According to previous research,^[^ ^35^, ^37^ ^]^ the ratio of the Pb_2_Au layer is Pb:Au = 2:1. The top is covered by Au layer, represented by the pink ball, which is commensurate with the middle Pb_2_Au layer and forms a Kagome structure, as shown in the center of Figure 1e. The unit cell marked by the black lines belongs to the Au Kagome structure, with the lattice constant of 5.7 Å observed in the STM image. Two lattices are superposed from the moiré pattern; one is Pb_2_Au superstructure consisting of Au Kagome layer and Pb_2_Au layer while the other is Pb(111). By adjusting the twist angle to 30°, we can obtain the moiré pattern where the lattice constant matches the STM image, as shown in the third part of Figure 1e.

Figure 1f shows STS spectra acquired from Pb(111) and Pb_2_Au superstructure at 4.8 K. All STS spectra show an obvious drop near the Fermi level, the superconducting gap. The black spectrum is measured on the bulk Pb(111); the red spectrum is measured on the bright spot of the Pb_2_Au superstructure; the blue spectrum is measured on the dark region. It can be seen that the zero‐bias conductance (ZBC) in the superconducting gap of the Pb_2_Au superstructure is deeper than that of the bulk Pb on the bright spot or the dark region. It is well known that the *T*_c_ of the bulk Pb_2_Au (≈3.15 K)^[^ ^38^ ^]^ is smaller than that of the bulk Pb, and the depth of the gap usually becomes smaller. This is due to the proximity effect that breaks down the Cooper‐pair correlation and turns a superconductor into a normal metal. Therefore, the STS spectra indicate the existence of the spontaneous superconductivity, which is even higher than that of the bulk Pb for the present 2D Pb_2_Au superstructure.

**Figure 2:**
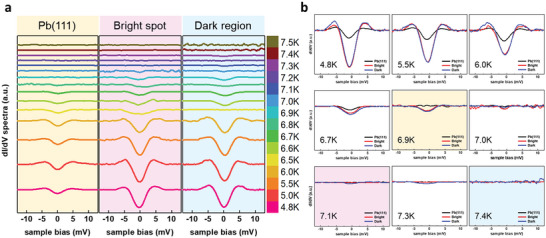
Temperature‐dependent STS spectra. a) Three series of the spectra labeled by light yellow, pink, and light blue background are acquired on Pb(111) bulk, bright spot of alloy layer, and dark region of alloy layer, respectively. b) Comparison of STS spectra from three series at the same temperature. The black curves represent Pb(111), the red curves represent the bright spot, and the blue curves represent the dark region, respectively.

**Figure** **2**a shows the temperature‐dependence d*I*/d*V* spectra measured within atomically flat terraces and selected temperatures within the range of 4.8 K ≤ *T* ≤ 7.5 K. The spectra are divided into three groups with different background color as light yellow, pink, and light blue. Spectral broadening can occur due to thermal fluctuations, particularly near the critical temperature. However, despite these fluctuations, each spectrum in Figure 2a represents an average of ten measurements taken during the experiment, allowing us to confidently judge the critical temperature. The STS spectra measured at temperatures from 4.8 with 0.1 K increasement each are shown with vertical shifts in Figure 2a. Each spectrum apparently shows the superconducting gap at the zero bias voltage. As expected, the ZBC increases as the temperature elevates, indicating a closing of the gap. Meanwhile, we have also measured STS for a clean Pb(111) surface as the reference value for subsequent measurements. The *T*_c_ of the clean Pb(111) surface is found to be 6.9 K, which is consistent to those determined from the middle point of the resistive phase transition (≈7.2 K).^[^ ^39^ ^]^ Focusing on the nearby temperature, the STS spectra of bright spot and dark region in the Pb_2_Au superstructure show closing of the superconducting gap at 7.1 and 7.4 K, respectively. Interestingly, the Pb_2_Au layer (both bright spot and dark region) reveals a lower ZBC than Pb(111) at the same temperature. This electronic phenomenon is more obvious when comparing three STS spectrums at the same temperature. As shown in Figure 2b, it is more distinct to see the disappearance of the superconducting gap at their respective *T*_c_.

In Figure 2b, black curves represent Pb(111) while red and blue curves represent the bright spot, and the dark region, respectively. At the initial temperature (4.8 K), the STS curves represented by all three colors reveal apparent gaps. As the temperature increases, the curves gradually become flatter. Once the temperature reaches 6.9 K, the black curve becomes horizontal, indicating that 6.9 K is the *T*_c_ of Pb(111). Accordingly, the gaps of the red and blue curves become imperceptible at 7.1 and 7.4 K, reflecting the *T*_c_ of bright spot and dark region, respectively. In addition, the *T*_c_ of Pb(111), bright spot, and dark region (6.9, 7.1, and 7.4 K) are marked with light yellow, light pink, and light blue background colors respectively to correspond to the counterparts in Figure 2a. Notably, the size of the superconducting gap Δ of Pb_2_Au, roughly approximated by the half of full width at half maximum, is 2.1 meV (the superconducting gap of bulk Pb is shown in the Supporting Information). With that, the ratio 2Δ/*k*_B_ *T*_c_ is approximated to be 6.9 and 6.6 (*T*_c_ are assumed as 7.1 and 7.4 K). Surprisingly, these constants are larger than the universal BCS ratio, 3.5, and are also different from 4.3 of bulk Pb,^[^ ^40^ ^]^ implying that there is a strong coupling mechanism in this 2D Pb_2_Au superstructure. Moreover, the *T*_c_ usually decreases when the thickness reduces,^[^ ^14^ ^]^ and it is inconsistent with the case we have observed here. In the BCS theory, $T_{c}=1.14\;T_{D}\;e^{-\;\frac{1}{VN(E_{f})}}$, the increase in *T*_c_ is strictly associated with DOS near *E*_F_, *N*(*E*_f_), and EPC potential, *V*. Therefore, revealing the electronic structures of Pb_2_Au superstructure can be a key for understanding the reason behind the current observation.

### 2.2 Rashba SOC in Buckled Pb_2_Au–Au Kagome Superstructure

**Figure 3:**
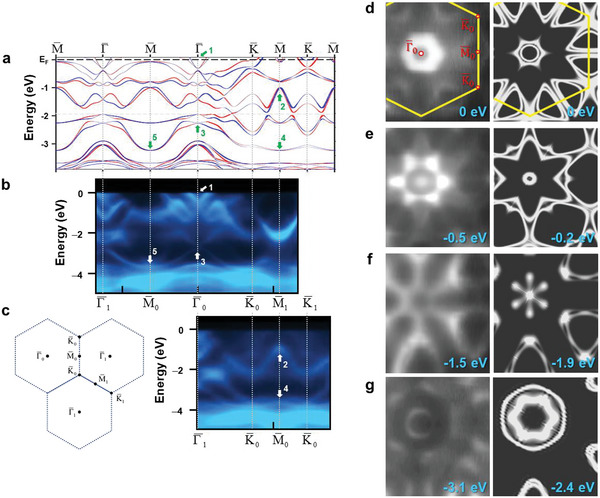
Electronic structures of buckled Pb_2_Au–Au Kagome superstructure. a) The calculated and b,c) measured energy band dispersions of the buckled Pb_2_Au–Au Kagome superstructure thin film in all symmetry directions. The red and blue colors indicate the in‐plane spin polarization in opposite directions. The arrows point out each Rashba splitting features. To achieve a better fit, the calculated *E*_F_ indicated by the gray dashed horizontal line is shifted to the black dashed horizontal line. d–g) 2D constant energy contours from the measurement (left column) and calculation (right column).

Chen et al. have investigated the electronic structures of Au deposited on both Pb thin films and bulk Pb(111).^[^ ^35^ ^]^ It was found that the first‐principles calculation on a single Pb_2_Au layer did not reproduce electronic band structures, measured by ARPES. Furthermore, they found a better resemblance when considering a Kagome Au layer on the top of the Pb_2_Au layer. Following the concept, **Figure** **3**a–c shows the new calculated and experimental energy band diagrams of the superstructure along all high symmetry directions. To consider the real material system, both the single Pb_2_Au layer and the top Kagome Au layer have contributed to the band structures. These structures collaborate with the bottom Pb(111) substrate to induce a buckle configuration of the sandwiched Pb_2_Au layer, as shown in Figure 1e. The DFT calculation first adopts the sandwich model using the three‐layer Pb(111) slab. The resulting buckle height of the middle Pb_2_Au layer is 1.5 Å. Then the band structure was calculated by considering the Pb_2_Au layer of this buckling configuration only with the Au Kagome layer on the top. The lattice constant employed is 5.7 Å as determined from STM and low‐energy electron diffraction (LEED).^[^ ^35^ ^]^ The experimental and theoretical LEED patterns are shown in the Supporting Information. It is noteworthy that this value indicates a ≈−5% lattice mismatch with underlying Pb(111)‐$\sqrt{3}\times \sqrt{3}R30^{\circ }$ lattice, which can be accounted for by the buckling‐induced contraction. The consequent band structures exhibit impressing Rashba effect as shown in Figure 3a. It is important to note that since the buckled Pb_2_Au–Au Kagome superstructure comprises two distinct layers, the orbital composition for each band structure is tangled. For example, the same band structure can be contributed from *p_x_p_y_* orbitals of the middle Pb_2_Au layer but from *p_z_* orbital of the top Au Kagome layer. Figure 3b,c extensively presents the experimental band structures along the symmetry directions in the surface Brillouin zone.

The salient features that indicate the correspondence between the calculation and measurement are labeled with numbers in Figure 3a–c. Feature 1 at the zone center ($\bar{\Gamma }$) presents a cone‐like Rashba splitting composed of a set of inner and outer bands. The composite bands appear to have different orbital parities so at the zone center of the first Brillouin zone (${\bar{\Gamma }}_{0}$), the cone structure has the most weight in the outer band. The 2D contours of Feature 1, shown in Figure 3d, appear as an inner circle and an outer hexagon from calculation while only the hexagon contour is resolved in the measured counterpart at ${\bar{\Gamma }}_{0}$. The hole pockets around $\bar{M}$ form ovals along each side of the hexagonal zone boundary. When the energy further goes down, as shown in Figure 3e, the cone contours at the center converge and the ovals along the zone boundary enlarge. Feature 2 indicates a *ω*‐shaped band but the calculation reveals that it is composed of bands with a slightly Rashba splitting at $\bar{M}$. In Figure 3f, the energies are intentionally chosen to show the contours of the *ω*‐shaped bands, exhibiting the consistent sector shape around $\bar{K}$ in both measurement and calculation. Feature 3 is a large Rashba splitting at the zone center, and the spin‐polarized composite bands of which extend throughout the surface Brillouin zone to join the Rashba splitting at $\bar{M}$ (Feature 4) in $\bar{\Gamma }\bar{K}\bar{M}$ direction and merge at $\bar{M}$ (Feature 5) in the $\bar{\Gamma }\bar{M}$ direction. It is worthwhile to notice that such an anisotropic Rashba splitting at the surface zone boundary $\bar{M}$ (Feature 4 and Feature 5) was also observed in a Bi‐trimer adlayer on a Si(111), similar to atomic layout of the top Kagome Au layer as shown in Figure 1e. In Figure 3g, the energies were intentionally chosen to show the energy contours of Feature 3 centered at the zone center ($\bar{\Gamma }$), revealing two hexagons 30° rotation from each other in both measurement and calculation. This indicates the warping effect in the Rashba splitting, which usually occurs in a system of threefold symmetry. Finally, the top Au Kagome layer greatly enhances Rashba effect and further modifies the DOS near *E*_F_,^[^ ^35^ ^]^ simultaneously triggering the enhancement of the *T*_c_ in the whole superstructure.

### 2.3 Enhance the *T*_c_ by Large EPC

**Figure 4:**
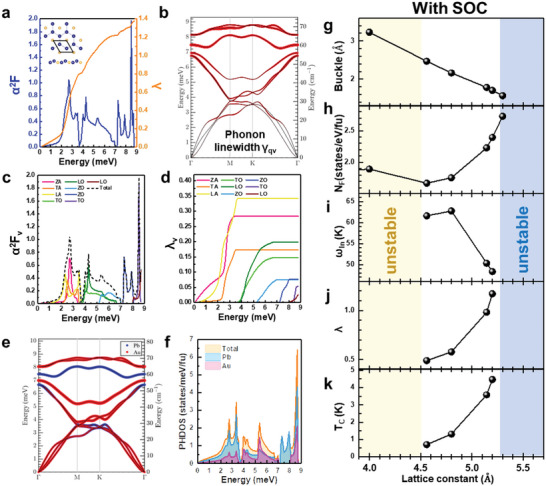
Electron–phonon coupling and superconductivity of Au‐plumbene. a) Total Eliashberg function (*α* ^2^ *F*) and EPC strength (*λ*) integrated from Eliashberg function. Inset: top view and side view of Pb–Au–Pb layer. The Pb atoms form the low‐buckled honeycomb plumbene structure with Au atom intercalated at the hollow site (Au‐plumbene). b) Phonon band dispersion and phonon linewidth (*γ_q*ν*_*) of each phonon mode *ν* at *q*‐point alone the high symmetry lines in Brillouin zone (BZ). The size of the red spheres denotes the phonon linewidth indicating the interaction intensity between electron and phonon, serving as the basic ingredient of EPC strength. c) Eliashberg function of each phonon mode integrated from the phonon linewidth over BZ. d) Energy‐integrated contribution of each phonon mode to the EPC strength. e) Phonon dispersion with the size of color spheres indicating the atomic contributions to the phonon mode. f) Atom‐decomposed phonon DOS. Note the red and blue curves denote phonon DOS for one Au atom and summing over 2 Pb atoms in the unit cell, respectively. (a–f) Lattice constant of 5.144 Å used in the DFT calculations. g) Buckling height, h) electronic DOS at the Fermi level, i) logarithmic average of phonon frequency, j) EPC strength, and k) superconducting critical temperature as functions of lattice constant. Yellow and blue regions indicate that the lattice structure is unstable with the lattice constant shorter than 4.5 Å or longer than 5.3 Å, respectively.

As shown in Figure 1e, the whole system is composed of three parts: the Pb(111) substrate, the Kagome Au cover‐layer, and the sandwiched Pb_2_Au layer. The Pb_2_Au layer can be considered as a plumbene layer with Au atoms inserted into the honeycomb centers, as shown in the inset of **Figure** **4**a, and will be denoted as Au‐Plumbene hereafter. It is worthwhile to notice that plumbene grown on Fe/Ir(111)^[^ ^22^ ^]^ also has a similar lattice constant to that of Au‐plumbene. The electronic DOS of Au‐plumbene using lattice constant of 5.14 Å with/without SOC and atom‐orbital decomposed band structure are shown in Figure S3 of the Supporting Information. We present the results based on the lattice constant 5.14 Å from Figure 4a–f because it leads to a relatively large *T*_c_ and is away from the unstable limit of 5.3 Å (will be discussed later). In order to show the spontaneous superconductivity of the Au‐plumbene structure, here we focus on the phonon calculation of the freestanding Au‐plumbene layer only. The calculation employing this simplified model shows the stable (positive) phonon band structure with lattice constant from 4.5 to 5.2 Å, whereas the phonon band structure becomes unstable when the lattice constant is smaller than 4.5 Å or exceeds 5.2 Å, at which the calculated *T*_c_ value is 4.7 K. For a better elucidation, we illustrated the case of lattice constant 5.14 Å and deduced that under the *T*_c_ of 3.8 K we are able to probe the mechanism of enhanced superconductivity.

The calculated total Eliashberg function (*α* ^2^ *F*) and EPC strength (*λ*) integrated from the Eliashberg function (see the Experimental Section) are shown in Figure 4a. The total *λ* = 1.4 of Au‐plumbene obtained in this work is notably stronger than *λ* = 1.2 (*E*_F_ = 0 eV) of pure plumbene studied in a previous work.^[^ ^28^ ^]^ Figure 4b shows the phonon band dispersion and phonon linewidth (*γ_q*ν*_*), which indicates the interaction intensity between electron and each phonon mode, serving as the basic ingredient of EPC strength. The phonon mode‐decomposed contributions to the Eliashberg function (the longitudinal, transverse, and the out‐of‐plane acoustic (optical) phonon modes marked as LA, TA, and ZA (LO, TO, and ZO)) and the integrated counterparts, as shown in Figure 4c,d, further demonstrate that two acoustic modes, especially the LA and ZA mode, contribute strongly to *α* ^2^ *F* in the low energy region ranging from zero to ≈3.5 meV. Three acoustic phonon bands contribute to the major part of *λ* ≈ 0.8. For the rest of the energy range, six optical phonon bands dominate and dedicate 0.4 to *λ* in the middle energy region (≈4 to ≈7 meV) and 0.2 to *λ* in the high energy region (≈7 to ≈9 meV), respectively.

To further explore the role of the intercalated Au and plumbene in Au‐plumbene, we recall the atomic contributions to the phonon band structures and phonon DOS in Figure 4e,f. Note the red and blue curves denote phonon DOS for one Au atom and the sum over 2 Pb atoms in the unit cell, respectively. As can be seen, the Au atom, which provides equally strong contribution as two Pb atoms, plays the important role in the three lower optical modes in the middle energy region, while Pb atoms dominate the phonon DOS in the low energy region, where *λ* is mainly contributed.

Finally, we summarize the buckling height, electronic DOS at the Fermi level (*N*_F_), logarithmic average of phonon frequency *ω*_ln_, EPC strength *λ*, and superconducting critical temperature *T*_c_ as functions of lattice constant in Figure 4g–k. Yellow and blue regions indicate that the lattice structure is unstable because of the calculated negative phonon bands with the lattice constant shorter than 4.5 Å or longer than 5.2 Å, respectively. Within the stable lattice structure range (white region), all the five physical quantities show consistent trends. The buckled height decreases along with the increasing lattice constant. The electron DOS increases due to the flattened band given by larger lattices. Although *ω*_ln_ decreases, which is not good for *T*_c_, the strongly enhanced *λ* and DOS combine together to enhance the superconducting *T*_c_ along with the increasing lattice constant as can be clearly seen in the figure. We note here that the experimental lattice constant of the whole sandwiched system shown in Figure 1e is 5.7 Å, and this value is totally out of the stable range in our simplified model without the Kagome Au cover‐layer (Figure 4a) for phonon calculations. Therefore, we assume that the on‐top Kagome Au cover‐layer and the Pb substrate can help stabilize the sandwiched Au‐plumbene with a larger lattice constant. The increasing *T*_c_ trend shown in Figure 4k might further enhance *T*_c_ up to the observed value in our experiments with a relatively larger lattice constant. On the other hand, the instability of Au‐plumbene toward the flat limit explains why a single flat Pb_2_Au binary layer has never been observed.^[^ ^35^ ^]^ Compared with the phonon structure of a pure plumbene,^[^ ^28^ ^]^ the Au‐plumbene has three additional optical phonon bands that contribute to EPC. Figure S4 of the Supporting Information provides evidences that both acoustic and optical phonon modes in out‐of‐plane directions contribute significantly to EPC, indicating the importance of buckling and intercalated Au in superconductivity of Au‐plumbene with a 2D honeycomb structure.

It is worth noting that as demonstrated from an In/Si(111)‐($\sqrt{7}\times \sqrt{3}$) atomic‐layer on a Si(111) surface,^[^ ^41^ ^]^ the Rashba effect may suppress Cooper pair‐breaking parameter by orders of magnitude and thereby supports superconductivity. It is also interesting to consider the Ising superconductivity with Zeeman‐type SOC.^[^ ^42^ ^]^ However, the calculated bands in Figure 3a do not reveal any features of Zeeman splitting except for Rashba‐type splitting. The measurement of in‐plane critical field for this plumbene system would be necessary in the future work.

**Figure 5:**
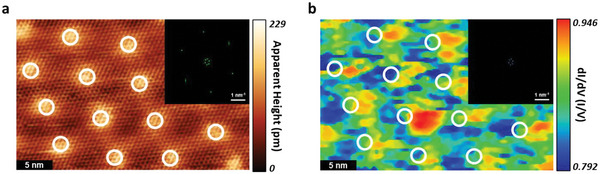
Spatial distribution of LDOS. a) The STM topographic image reveals a moiré pattern with a potential modulation. The bright spots are marked by white circles. b) d*I*/d*V* mapping is obtained in the same area as (a). The correlated conductance distribution can be seen (see text). The imaging condition is *V* = −0.5 V, *I* = 1.0 nA, and image size are 20 × 33 nm^2^. The FFT images are shown in the insets. Both the STM image and the d*I*/d*V* mapping exhibit the inner six spots in the individual FFT images, indicating the existence of the same periodicity as the moiré pattern.

While the modification of buckled height, electronic DOS, phonon frequency, and EPC explain the enhancement of *T*_c_ in buckled plumbene‐Au Kagome superstructure, the spatial distribution of LDOS in two dimensions may also give rise to another modulation for superconducting behavior.^[^ ^43^ ^]^ As mentioned in Figure 2, a difference in *T*_c_ is found between bright spots and dark regions on the moiré pattern of the superstructure (7.1 and 7.4 K, respectively). Therefore, we further investigated the local DOS distribution on the buckled plumbene‐Au Kagome superstructure surface. In **Figure** **5**, the d*I*/d*V* mapping (Figure 5b) was obtained simultaneously with the topography of Figure 5a. The topography shows the moiré period as mentioned in Figure 1, and the white circles mark the modulated pattern that appears periodically. The d*I*/d*V* mapping in Figure 5b reveals a similar periodicity, as confirmed by the corresponding FFT inset images displaying the inner six spots. These spots represent the moiré pattern (as described in Figure 1d), while the outer six spots represent the basic 1 × 1 atomic configuration, which is observable only in the STM image. The periodical moiré potential modulates the electron density near the Fermi level and this modulation can affect both the strength of SOC and LDOS, resulting in a difference in *T*_c_ on the buckled plumbene‐Au Kagome superstructure. As a consequence, our results suggest that moiré potential can further modulate superconducting behavior in the 2D limit.

## 3 Conclusion

In conclusion, we discovered the enhanced superconductivity in a buckled plumbene‐Au Kagome superstructure grown on the Pb(111) surface, in which *T*_c_ is much higher than any monolayer Pb. By temperature‐dependent STS measurements, the *T*_c_ of Pb(111), and the superstructure at the bright spot, and dark region are determined to be 6.9, 7.1, and 7.4 K, respectively, showing a distinct increment in *T*_c_. The superstructure is considered to be a monolayer Au‐intercalated low‐buckled plumbene (Au‐plumbene) sandwiched by a top Au Kagome layer and bottom Pb(111), as confirmed by measured and calculated electronic structures; a large Rashba effect is further unveiled. DFT calculations reveal strong EPC in this Au‐plumbene layer, leading to the enhanced *T*_c_. The Au atoms situated in the plumbene honeycomb centers provide an important example of triggering novel properties of 2D materials in honeycomb lattice. The coexistence of large Rashba effect and electron–phonon enhanced superconductivity makes the buckled plumbene‐Au Kagome superstructure an idealistic system to control the physical properties of the 2D layer in exotic ways for designing functional and novel materials such as 2D topological superconductors in the application of fundamental research and new quantum engineering.

## 4 Experimental Section

### Sample Preparation

The synthesis of buckled plumbene‐Au Kagome superstructure was achieved by depositing Au atoms on Pb(111) in an ultrahigh vacuum with a base pressure of 5 × 10^−10^ Torr. The polished Pb(111) single crystals from MaTecK were used in this work. The Pb(111) substrate was cleaned by a standard cycle of Ar^+^ ions bombardment (1 keV) followed by thermal annealing at 130 °C. Au was evaporated from an alumina crucible in a Knudsen cell at 1050 °C. The vacuum pressure was lower than 8 × 10^−9^ Torr during deposition and the deposition rate was controlled at 1 ML h^−1^. During the process of deposition, the Pb single crystal was kept at room temperature.

### STM and STS Experiments

The buckled plumbene‐Au Kagome superstructure was characterized by an Omicron STM with a base pressure of 5 × 10^−11^ Torr. A tungsten tip was used for STM and STS measurements. The STS measurements were acquired by using a lock‐in amplifier with a small sinusoidal modulation to the sample bias voltage (0.4 mV, 520 Hz). The correctness of STS was verified by checking the standard d*I*/d*V* spectrum of Au(111) and Pb(111) with the same tip. All STM and STS measurements were performed at 4.8 to 7.5 K.

### ARPES Experiments

The angle‐resolved photoemission spectra were measured with a Scienta R4000 energy analyzer using a *p*‐polarized light source at 22 eV at the undulator beamline BL21B1 at the National Synchrotron Radiation Research Center in Taiwan. The energy and angular resolutions were 10 meV and 0.3°.

### DFT Calculations

DFT calculations were performed using the Vienna ab initio simulation package^[^ ^44^, ^45^, ^46^, ^47^ ^]^ with the projector‐augmented‐wave pseudopotential^[^ ^48^, ^49^ ^]^ utilizing the Perdew–Burke–Ernzerhof exchange‐correlation functional. Plane wave basis with the energy cutoff of 400 eV was used in the self‐consistent‐field calculations over the 12 × 12 × 1 k‐point mesh in the 2d Brillouin zone. The spin–orbit coupling was included in the self‐consistent calculations of electronic structure. The Kagome Au/Au‐plumbene/7ML Pb(111) sandwich model as shown in Figure 1e was first adopted to simulate the experimental system. After the atomic‐coordinate relaxation with the experimental lattice constant of 5.7 Å, the 7 ML Pb(111) substrate was then removed, leaving the freestanding Kagome Au/Au‐plumbene bilayer structure with the buckle height of 1.5 Å in the plumbene layer. To best fit the ARPES results, the Au atom within the plumbene layer was shifted downward by 0.6 Å.

For lattice dynamic and superconductivity calculation, the Quantum‐Espresso code^[^ ^50^, ^51^ ^]^ based on the density functional perturbation theory (DFPT)^[^ ^52^ ^]^ and Allen‐Dynes modify McMillan Formula was used.^[^ ^53^, ^54^ ^]^ Ultrasoft pseudopotential with spin–orbital coupling was adopted in the DFPT calculations. Energy cutoff of 60 and 600 Ry was used for kinetic wavefunction and charge density, respectively. Because DFPT calculations for the previously described sandwich model were not feasible owing to the highly demanding computing resources, thus the freestanding Kagome Au/Au‐plumbene bilayer and Au‐plumbene systems instead were studied. The former case showed negative phonon bands and was unstable. Thus the freestanding Au‐plumbene layer hereafter was kept on observation. The Au‐plumbene lattice structure was first optimized until the atomic residual forces were less than 10^−5^ Ry a.u.^−1^. Then DFPT calculations were performed using 12 × 12 × 1 k‐mesh and 6 × 6 × 1 q‐mesh for phonon dispersion. The electron–phonon coupling constant *λ* is calculated using the 24 × 24 × 1 k‐mesh interpolated over the Brillouin zone via $\lambda \left(\omega \right)=\int_{0}^{\omega }d\omega^{\prime }\frac{2\alpha^{2}F\left(\omega^{\prime }\right)}{\omega^{\prime }}$. The Eliashberg function *α* ^2^ *F*(*ω*) is calculated by $\alpha^{2}F\;\left(\omega \right)=\frac{1}{N_{F}}\;\int_{\mathrm{BZ}}\frac{dkdq}{\Omega_{\mathrm{BZ}}}\sum_{m,n,\nu }{|g_{mn\nu }\left(k,q\right)|}^{2}\delta \left(\varepsilon_{n,k}-\varepsilon_{F}\right)\delta \left(\varepsilon_{m,k+q}-\varepsilon_{F}\right)\delta \left(\omega -\omega_{q\nu }\right)$, where *N*_F_ is density of states at Fermi level, *g*_*mnν*_ is electron–phonon matrix element, *ε*_*n*,*k*_ is electronic band at number of *n* and wavenumber *k*, *ε*_F_ is Fermi level, *ω*_*qν*_ is phonon frequency at number of *ν* and wavenumber *q*, and Ω_BZ_ is Brillouin zone volume. The critical temperature *T*_c_ of superconductivity is estimated by the Allen–Dynes modified McMillan formula $T_{c}=\frac{\omega_{\ln}}{1.2}\;\exp\left(\frac{-1.04(1+\lambda )}{\lambda -\mu^{*}\left(1+0.62\lambda \right)}\right)$, where *λ* is total electron–phonon coupling constant, µ* = 0.1 is the effective Coulomb repulsion,^[^ ^55^ ^]^ and *ω*_ln_ is logarithm average phonon frequency: $\omega_{\log}=\exp\left[\frac{2}{\lambda }\int d\omega \frac{\ln\omega }{\omega }\alpha^{2}F\left(\omega \right)\right]$.

## Conflict of Interest

The authors declare no conflict of interest.

## Supporting information

Supporting Information

## Data Availability

The data that support the findings of this study are available from the corresponding author upon reasonable request.
