# Supplementary material for: Enhanced Superconductivity and Rashba Effect in a Buckled Plumbene‐Au Kagome Superstructure
Source: Adv Sci (Weinh). 2023 May 3;10(17):2300845. doi: 10.1002/advs.202300845 (PMC10265087; doi:10.1002/advs.202300845)
Supplement: Supplementary file 1 — Supporting Information [file ADVS-10-2300845-s001.pdf]

ADVANCED SCIENCE
Open Access

Supporting Information

for *Adv. Sci.*, DOI 10.1002/advs.202300845

Enhanced Superconductivity and Rashba Effect in a Buckled Plumbene-Au Kagome Superstructure

Wan-Hsin Chen, Chin-Hsuan Chen, Guan-Hao Chen, Wei-Chuan Chen, Fu-Xiang Rikudo Chen, Pei-Jung Chen, Chun-Kai Ku, Chang-Tsan Lee, Naoya Kawakami, Jia-Ying Li, Iwao Matsuda, Wen-Hao Chang, Juhn-Jong Lin, Chien-Te Wu, Chung-Yu Mou, Horng-Tay Jeng, Shu-Jung Tang* and Chun-Liang Lin**

Supporting Information

Enhanced Superconductivity and Rashba Effect in a Buckled Plumbene-Au Kagome Superstructure

Wan-Hsin Chen[1†], Chin-Hsuan Chen[2†], Guan-Hao Chen[1,3,4†], Wei-Chuan Chen[2], Fu-Xiang Rikudo Chen[1], Pei-Jung Chen[1], Chun-Kai Ku[1], Chang-Tsan Lee[1], Naoya Kawakami[1], Jia-Ying Li[2], Iwao Matsuda[5], Wen-Hao Chang[1,3,4], Juhn-Jong Lin[1,4], Chien-Te Wu[1], Chung-Yu Mou[2], Horng-Tay Jeng[2,6,7], Shu-Jung Tang[2,8*], Chun-Liang Lin[1*]*

[1]Department of Electrophysics, National Yang Ming Chiao Tung University, Hsinchu 300, Taiwan.
[2]Center for Quantum Technology and Department of Physics, National Tsing Hua University, Hsinchu 300, Taiwan.
[3]Research Center for Applied Sciences, Academia Sinica, Taipei 115, Taiwan.
[4]Center for Emergent Functional Matter Science, National Yang Ming Chiao Tung University, Hsinchu 300, Taiwan.
[5]Institute for Solid State Physics, The University of Tokyo, Kashiwa 277-8568, Japan.
[6]Physics Division, National Center for Theoretical Sciences, Taipei 106, Taiwan.
[7]Institute of Physics, Academia Sinica, Taipei 115, Taiwan.
[8]National Synchrotron Radiation Research Center, Hsinchu 300, Taiwan.

* Corresponding author. Email: clin@nycu.edu.tw, sjtang@phys.nthu.edu.tw, jeng@phys.nthu.edu.tw
†These authors contributed equally to this work.

STS spectra and superconducting gap

Figure 1S shows a (d*I*/d*V*)/(*I*/*V*) normalized STS spectra obtained on Pb(111) at 4.8 K. The superconducting gap is observed at around the E_F. The red curve is a fitting result of the Dynes function[1]:

$$\frac{dI(V)}{dV} \propto \int_{-\infty}^{\infty} dEN_s(E)\left(-\frac{\partial f(E-eV)}{\partial \mathrm{V}}\right)$$

where

$$N_s(E) = E_n(E_F)Re\frac{E-i\Gamma}{\sqrt{(E-i\Gamma)^2-\Delta^2}}$$

(Δ is the superconducting gap and Γ is Dyne's broadening factor). From the fitting result and the full width at half maximum (FWHM) between the two conductance peaks, we suggest that the superconducting energy gap Δ is about 2.9 and 1.9 meV, respectively. Even though the value is larger than the Pb bulk value $\Delta_{Bulk\ Pb}$[3.0 K] = 1.23 meV (ref. [2]), it does not affect the T_c in comparison with that of the Pb_2Au superstructure because the superconducting gap always disappears at T_c. In order to make the gap decision process simpler, we compare the gap size difference between Pb and Pb_2Au superstructure by FWHM.

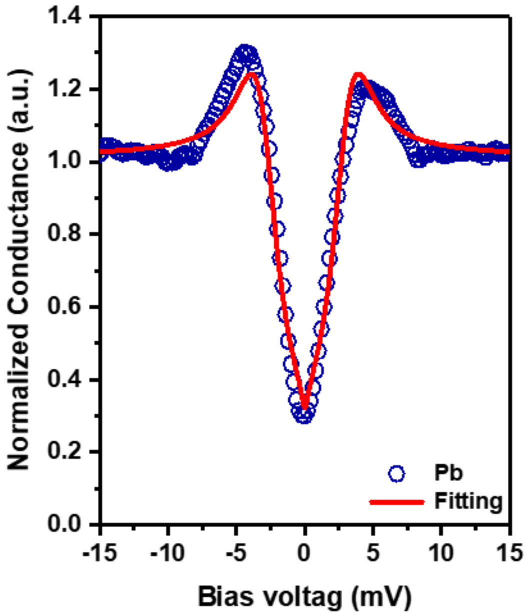

Figure S1. The STS spectra of Pb(111) obtained at T = 4.8 K (blue circles). The resolution is 0.2 mV and the lock-in modulation *Vrms* = 400 μV at a frequency of 520 Hz. The red solid curve is the fitting of the Dynes function.

LEED patterns

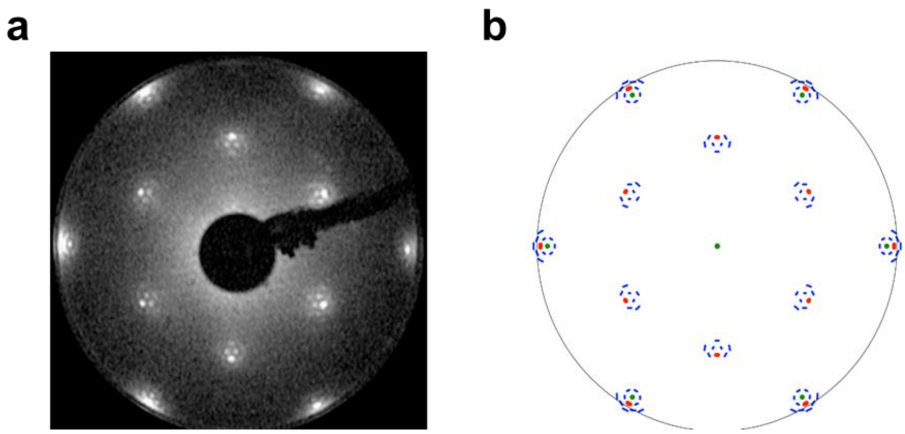

Figure S2. a) LEED pattern of the buckled plumbene-Au Kagome superstructure on Pb(111) taken at 40 eV. b) The simulation of the LEED pattern. The green and red circles are the single-scattering spots from Pb(111) and PbAu alloy layer. Others are the double-scattering spots. The resulting lattice structure is Pb(111)-$0.95\sqrt{3} \times 0.95\sqrt{3}R30°$ with lattice constant 5.7Å.

.

Electronic density of states and atom-orbital-decomposed band structure

The electronic density of states (DOS) of Au-Plumbene using lattice constant of 5.144 Å with/without spin-orbit coupling (SOC) are shown in Figure S3a. Overall, the DOS with/without SOC differ from each other significantly. But near the Fermi level, they are similar to each other. Owing to the strong SOC in both Pb and Au, here we only discuss results with SOC included in all the other figures in Figure 4. Figure S3b shows the atom-orbital-decomposed band structure. The two bands crossing the Fermi level are mainly composed of Pb-p orbitals with minor contributions from Au-d_{xz}-d_{yz} orbitals around the K point. These two bands form a small hole pocket at the zone center and a large electron pocket circulating around the zone boundary.

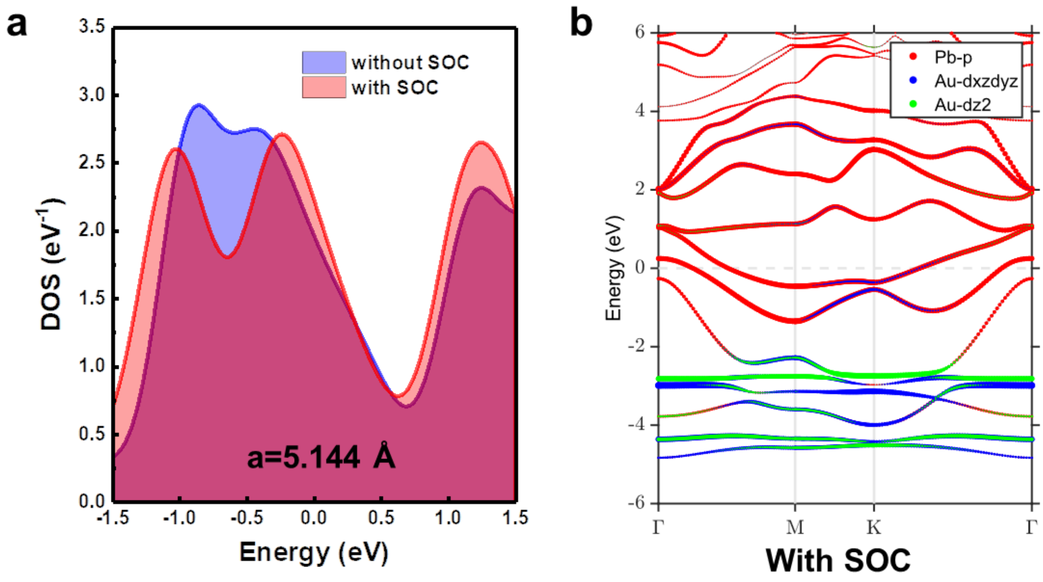

Figure S3. a) Density of states of Au-Plumbene with/without SOC using lattice constant a=5.144 Å. This lattice constant is also used for Figure 4a to 4f. b) Atom-orbital decomposed band structure with spin-orbit coupling (SOC) included.

The vibration forms of the phonon branches

The vibration forms of the nine phonon branches (Figure S4, b and c), same as Figure 4e at the Γ point are shown in Figure S4, d-f). The labels of these phonon modes denote the type of the phonons. A and O indicate the acoustic and optical modes, respectively. L stands for longitudinal waves, T for in-plane transversal waves, and Z for out-of-plane transversal waves. The phonon vibrations and the energy order: ZA, TA, LA of the three acoustic modes shown in Figure S4d are the same as those of other two-dimensional materials such as graphene.

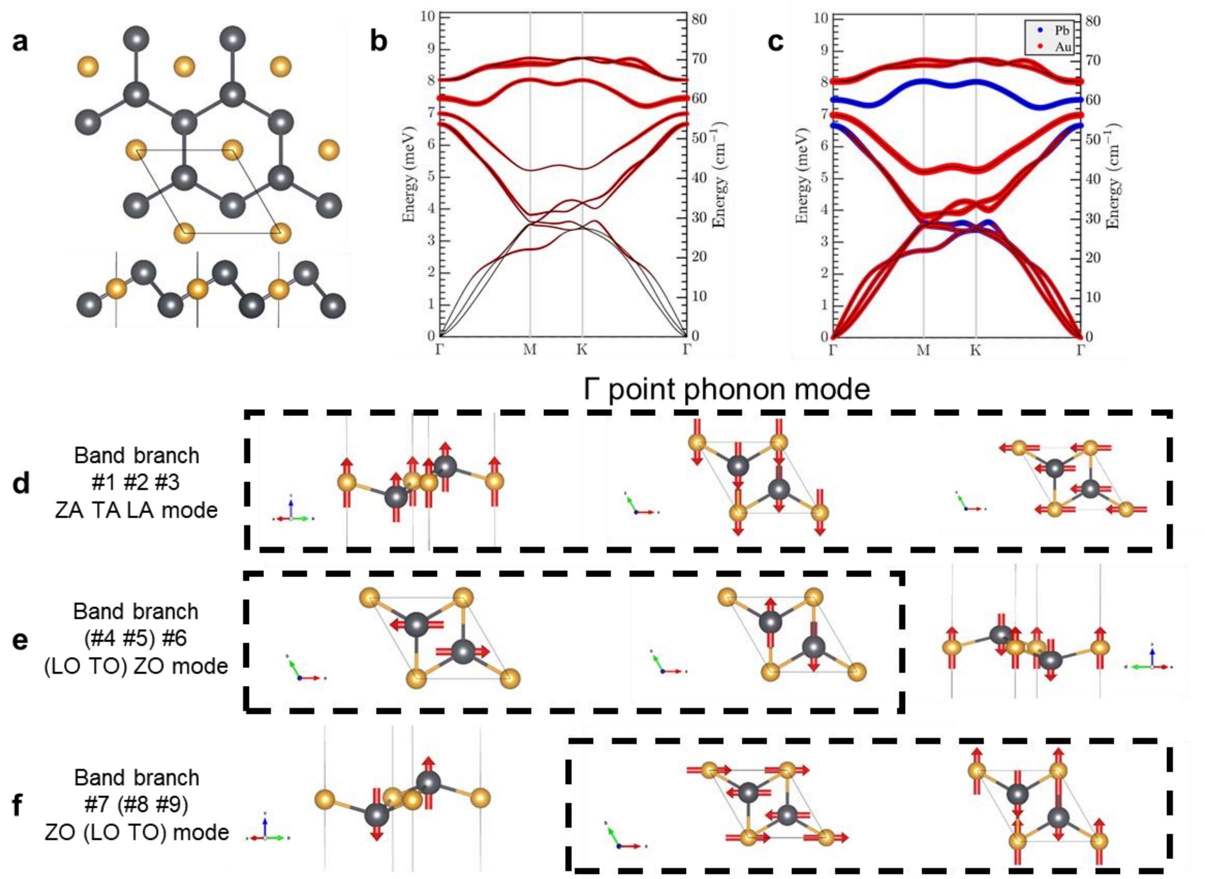

Figure S4. a) Top view and side view of Pb-Au-Pb layer. The Pb atoms form the buckled honeycomb Plumbene structure with Au atom intercalated at the hollow site. b) Phonon dispersion with the size of red spheres indicating the Eliashberg function. c) Phonon dispersion with the size of colored spheres indicating the atomic contributions to the phonon mode. Figure S3 a), b), c) are the same as Figure 4 a), b), e), respectively. d) Phonon branch #1-3 of ZA, TA, LA mode at Γ point, respectively. e) Phonon branch #4-6 of LO, TO, ZO mode at Γ point, respectively. f) Phonon branch #7-9 of ZA, TA, LA mode at Γ point, respectively. The dashed rectangular indicates the degenerated modes.

Kagome bands

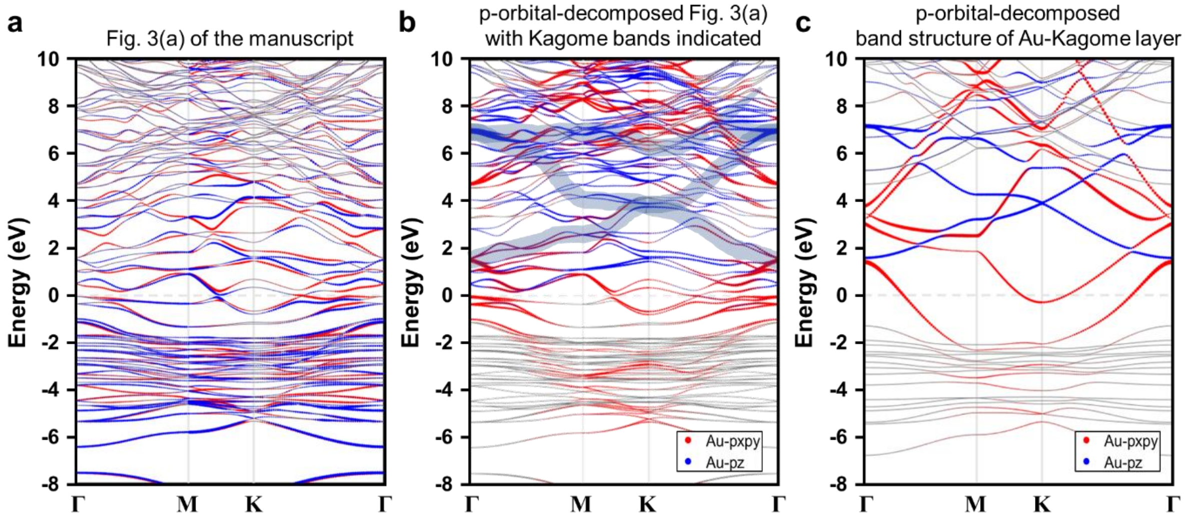

Figure S5. a) In-plan-spin-decomposed band structure of Kagome Au/Au-Plumbene. This figure is the same as Figure. 3 a) in the maintext with a wider energy range for indicating the Kagome bands. The dashed and dash-dotted horizontal lines indicate the calculated and measured Fermi level, respectively. b) p-orbital-decomposed band structure of Kagome Au/Au-Plumbene with the Kagome bands from the Kagome Au layer indicated by the grey-shaded bands. The Au-pz character (blue) of the flat band around 7 eV near the Γ-point and the Dirac point around 3.8 eV at the K-point are clearly captured. c) p-orbital-decomposed band structure of freestanding Kagome Au layer. The pz bands (blue) form the typical topological Kagome bands with the Dirac point around 3.7 eV at the K-point and the flat band around 7 eV. The pz flat band interacts strongly with pxpy bands around K point, resulting in notable band splittings.

1. N. Bergeal, V. Dubost, Y. Noat, W. Sacks, D. Roditchev, N. Emery, C. Hérold, J-F. Marêché, P. Lagrange, G. Loupias, Scanning tunneling spectroscopy on the novel superconductor. *Phys. Rev. Lett.* **2006**, *97*, 077003.
2. C. Brun, I.-P. Hong, F. Patthey, I. Y. Sklyadneva, R. Heid, P. M. Echenique, K. P. Bohnen, E. V. Chulkov, W.-D. Schneider, Reduction of the superconducting gap of Ultrathin PB islands grown on Si(111). *Phys. Rev. Lett.* **2009**, *102*, 207002.
